# Integrated analyses reveal IDO1 as a prognostic biomarker coexpressed with PD-1 on tumor-associated macrophages in esophageal squamous cell carcinoma

**DOI:** 10.3389/fphar.2024.1466779

**Published:** 2024-09-16

**Authors:** Yaojun Peng, Lingxiong Wang, Juan Yang, Qiyan Wu, Xiaoxuan Sun, Jinying Zhang, Yanju Yu, Liping Zhang, Jie Gao, Qing Zhou, Haiyan Zhu, Fan Yin

**Affiliations:** ^1^ Department of Emergency, The First Medical Center, Chinese People’s Liberation Army (PLA) General Hospital, Beijing, China; ^2^ Medical School of Chinese People’s Liberation Army (PLA), Beijing, China; ^3^ Lab of the Oncology Department, The First Medical Center, Chinese People’s Liberation Army (PLA) General Hospital, Beijing, China; ^4^ Institute of Oncology, The Fifth Medical Centre, Chinese People’s Liberation Army (PLA) General Hospital, Beijing, China; ^5^ Department of Cardiothoracic Surgery, Tianjin Fourth Center Hospital, Tianjin, China; ^6^ National Clinical Research Center for Cancer, Key Laboratory of Cancer Prevention and Therapy, Tianjin’s Clinical Research Center for Cancer, Tianjin Medical University Cancer Institute and Hospital, Tianjin, China; ^7^ Department of Oncology Surgery, Tianjin Cancer Hospital Airport Free Trade Zone Hospital, Tianjin, China; ^8^ Department of Basic Medicine, Medical School of Chinese People’s Liberation Army (PLA), Beijing, China; ^9^ Department of Oncology, The Second Medical Center and National Clinical Research Center of Geriatric Disease, Chinese People’s Liberation Army (PLA) General Hospital, Beijing, China; ^10^ Department of Gastroenterology, The Second Medical Center and National Clinical Research Center of Geriatric Disease, Chinese People’s Liberation Army (PLA) General Hospital, Beijing, China

**Keywords:** immune checkpoint, esophageal squamous cell carcinoma, macrophage, immune microenvironment, PD-1, IDO1

## Abstract

**Background:**

Inhibition of indolamine-2,3-dioxygenase 1 (IDO1) has been proposed as a promising strategy for cancer immunotherapy; however, it has failed in clinical trials. Macrophages in the tumor microenvironment (TME) contribute to immune escape and serve as potential therapeutic targets. This study investigated the expression pattern of IDO1 in TME and its impact on prognosis and therapeutic response of patients with esophageal squamous cell carcinoma (ESCC).

**Methods:**

RNA sequencing data from 95 patients with ESCC from The Cancer Genome Atlas (TCGA) database were used to explore the prognostic value of IDO1. Bioinformatics tools were used to estimate scores for stromal and immune cells in tumour tissues, abundance of eight immune cell types in TME, and sensitivity of chemotherapeutic drugs and immune checkpoint (IC) blockage. The results were validated using digitalized immunohistochemistry and multiplexed immunofluorescence in ESCC tissue samples obtained from our clinical center.

**Results:**

TCGA and validation data suggested that high expression of IDO1 was associated with poor patient survival, and IDO1 was an independent prognostic factor. IDO1 expression positively correlated with macrophages in TME and PDCD1 within diverse IC genes. Single-cell RNA sequencing data analysis and multiplexed immunofluorescence verified the coexpression of IDO1 and PD-1 in tumor-associated macrophages (TAMs). Patients with high IDO1 expression showed increased sensitivity to various chemotherapeutic drugs, while were more likely to resist IC blockage.

**Conclusion:**

This study identifies IDO1 as an independent prognostic indicator of OS in patients with ESCC, reveals a compelling connection of IDO1, PD-1, and TAMs, and explores the sensitivity of patients with high IDO1 expression to chemotherapeutic drugs and their resistance to IC blockade. These findings open new avenues for potential targets in ESCC immunotherapy.

## Introduction

Esophageal cancer is a major public health concern worldwide, with a poor prognosis (Sung et al., 2021). Esophageal squamous cell carcinoma (ESCC) is the predominant histological subtype of esophageal cancer, accounting for approximately 90% of all cases. ESCC has a unique etiology, molecular profile, and clinicopathological features (Rustgi and El-Serag, 2014; Ooki et al., 2023). Many patients are diagnosed at an advanced stage, delaying treatment initiation (Liang et al., 2017). Despite the implementation of early cancer screening strategies and multimodal treatments, the high incidence and low survival rate of ESCC remain urgent issues that need to be addressed (Fong et al., 2020). Therefore, it is important to identify novel molecular markers and develop effective therapeutic strategies.

The development and approval of immune checkpoint (programmed cell death protein 1 [PD-1], programmed cell death-ligand 1 [PD-L1], and cytotoxic T-lymphocyte associated protein 4 [CTLA4]) inhibitors leaded to dramatic changes in the landscape of cancer therapy (Robert, 2020). However, only a small proportion of patients exhibit long-lasting responses, and most patients treated with an immune checkpoint inhibitor, especially with a monotherapy approach, will demonstrate either primary or acquired resistance (Sharma et al., 2017). Multiple mechanisms of resistance have been proposed, among which L-tryptophan (Trp) catabolism was suggested as a critical contributor (Fujiwara et al., 2022).

Trp is an essential amino acid, playing a vital role in cell growth and protein synthesis (Badawy, 2017). Trp generally participants in three main metabolic processes: production of proteins, incorporation into serotonin anabolism, and transformation into kynurenine (Kyn) (Platten et al., 2012). Three enzymes (indoleamine 2,3-dioxygenase [IDO] 1, IDO2, and tryptophan 2,3-dioxygenase [TDO]) catalyze the rate-limiting step of the Kyn pathway. In particular, IDO1 is overexpressed and constitutes a poor prognostic marker in many tumours including endometrial cancer (Ino et al., 2006), laryngeal squamous cell carcinoma (Ye et al., 2013), melanomas (Speeckaert et al., 2012), gastric cancer (Li et al., 2019), hepatocarcinoma (Pan et al., 2008), and cervical cancer (Inaba et al., 2010). As for esophageal cancer, IDO1 was shown to be associated with immune tolerance and poor prognosis in patients with surgically resected tumours (Kiyozumi et al., 2019a). Zhou et al. (2020) revealed that IDO1 and PD-L1 expression and CD8 density increased significantly after neoadjuvant chemoradiation therapy in ESCC, and could serve as prognostic biomarkers for survival. IDO1 promoter hypomethylation was found to regulate its mRNA upregulation in esophageal cancer (Kiyozumi et al., 2019b). Mechanically, IDO1 facilitated esophageal carcinoma progression by driving the direct binding of NF-κB and CXCL10 (Yao et al., 2023). Nonetheless, the link between IDO1 and ESCC has not yet been fully elucidated, and further research on its role in the tumor microenvironment (TME) is needed. Therefore, this study aimed to investigate the expression pattern of IDO1 in TME and its impact on prognosis and therapeutic response of patients with ESCC based on publicly available datasets and experimental validation in ESSC samples from our clinical center.

## Materials and methods

### TCGA ESCC dataset and ESCC tissue samples

The RNA sequencing data for 95 newly diagnosed ESCC patients from the TCGA database (https://cancergenome.nih.gov/) were downloaded from the UCSC Xena platform (https://xenabrowser.net/datapages/). Tumour samples were collected before chemotherapy, targeted therapy, or radiotherapy. Clinicopathologic characteristics, including age, sex, tumour grade, TNM stage, overall survival (OS) time, and survival status, were obtained (summarized in Supplementary Table S1). The TCGA ESCC dataset was designated as the discovery cohort.

Formalin-fixed paraffin-embedded (FFPE) tissue samples were collected from 77 patients with ESCC after surgical resection at the First Medical Center of the Chinese PLA General Hospital between 18 July 2010, and 27 May 2011. These patients did not undergo chemotherapy, targeted therapy, or radiotherapy before surgical resection. Patients’ clinicopathologic characteristics were summarized in Supplementary Table S1. This group of patients was designated as the validation cohort. This study was approved by the Ethics Committee of the Chinese PLA General Hospital (No. S2019-228-02). All experimental procedures were performed in accordance with the principles of the Declaration of Helsinki.

### Survival analysis

A total of 78 immunomodulators was retrieved from a published study (Thorsson et al., 2018). These immunomudulators were classified into seven categories: antigen presentation, cell adhesion, co-inhibitors, co-stimulators, ligands, receptors, and others (summarized in Supplementary Table S2). The expression of these immunomodulators was extracted from the mRNA matrix of TCGA ESCC, and integrated with their corresponding survival information. Univariate Cox regression analysis was performed to identify immunomodulators with prognostic value utilizing the “survival” package of R software (version 4.1.2).

The prognostic value of IDO1 in patients with ESCC was evaluated using the Kaplan-Meier method and the log-rank test. The “maxstat” R package was used to calculate the best cutoff point of IDO1 expression for subgrouping patients. The minimum sample size for low- and high-IDO1 subgroups was set as more than 25% of the total count of the entire cohort. A Cox proportional hazards model was used to explore whether IDO1 was an independent determinant of OS in patients with ESCC.

### Evaluation of tumour purity

The ESTIMATE algorithm computes scores for the infiltration of stromal and immune cells in tumour tissues based on RNA-seq data (Yoshihara et al., 2013). Stroma, immune, and ESTIMATE scores of TCGA ESCC samples were retrieved from the ESTIMATE website, (https://bioinformatics.mdanderson.org/estimate/).

### Estimation of immune cell infiltration

The abundance of eight types of cells (B cells, cancer-associated fibroblasts [CAFs], CD4 T cells, CD8 T cells, endothelial cells, macrophages, natural killer [NK] cells, and uncharacterized [UC] cells) in TME of TCGA ESSC estimated by the EPIC method (Racle et al., 2017) was downloaded from the TIMER2.0 website (http://timer.comp-genomics.org/) (Li et al., 2020).

### Analysis of single-cell RNA sequencing data

The IMMUcan scDB database (https://immucanscdb.vital-it.ch/) is an accessible and supportive tool for deciphering tumour-associated single-cell RNA sequencing (scRNA-seq) data, allowing researchers to maximize the use of these data to provide new insights into cancer biology (Camps et al., 2023). scRNA-seq datasets of ESCC were searched within this database. The cell type compositions of each sample were explored by selecting the “UAMP Plot” panel with a given annotation, such as immune cell type assignment. The “Gene X vs. Gene Y expression” panel was used to evaluate gene coexpression at the cell type resolution.

### *In silico* prediction of therapeutic response

The sensitivity of 13 commonly used chemotherapeutic medications (cisplatin, docetaxel, doxorubicin, gefitinib, gemcitabine, nilotinib, paclitaxel, rapamycin, roscovitine, sorafenib, sunitinib, vinblastine, and vorinostat) was evaluated using the “pRRophetic” R package (Geeleher et al., 2014b; Geeleher et al., 2014a). The half-maximal inhibitory concentration (IC50) was an indicator of the response rate of chemotherapeutic drugs. Additionally, we employed the Tumour Immune Dysfunction and Exclusion (TIDE) algorithm (Jiang et al., 2018) (http://tide.dfci.harvard.edu/) to evaluate T-cell dysfunction and exclusion in TCGA ESCC to infer their therapeutic response to immune checkpoint (IC) blockade.

### Pathway enrichment

Metascape is a well-recognized and web-based tool for gene annotation (http://metascape.org) (Zhou et al., 2019), which was employed to explore signaling pathways associated with IDO1 in this study. Firstly, differentially expressed genes (DEGs) between the low- and high-IDO1 subgroups were screened using the “limma” R package. The filtering criteria were set as log_2_ |fold change| ≥ 1 and adjusted *P*-value <0.05. The DEGs obtained in the first step were then subjected to Metascape for Gene Ontology (GO) and Kyoto Encyclopedia of Genes and Genomes (KEGG) analyses. All genes in the genome were used as the enrichment background. Terms with a *P*-value less than 0.01, a minimum count of three, and an enrichment factor (defined as the ratio between the observed counts and the counts expected by chance) greater than 1.5 were selected. Finally, terms that were significantly enriched in the second step were grouped into clusters based on their membership similarities, and the most statistically significant term within each cluster was selected to represent the cluster.

### Immunohistochemistry and digital pathology assessment

Four-micrometer sections were prepared from the FFPE samples. The sections were deparaffinized and rehydrated using graded ethanol. The sections were then subjected to heat-induced epitope retrieval using a citrate buffer solution. Endogenous peroxidase activity was quenched using 0.3% H_2_O_2_ and non-specific binding was achieved by blocking with 5% goat serum. Next, the sections were incubated with an anti-IDO1 antibody (1:800, CST, #86630) at 4°C overnight, followed by incubation with a secondary antibody for 30 min at 37°C. IDO1 staining was visualized with 3,3′-diaminobenzidine (DAB, ZSGB-BIO, ZLI-9018) and counterstained with hematoxylin.

An Olympus SLIDEVIEW VS200 research slide scanner was used to capture images of the stained slides, and these pathological images were rendered in a whole-slide image (WSI) format. The quantitative analysis of all WSIs was performed using QuPath (version 0.4.2) (Bankhead et al., 2017), as previously described in Zheng et al. (2022). In brief, the watershed cell detection method was used to identify and segment cells in a slide. Representative specific regions were manually selected to classify tumour cells and stroma cells. A random tree classifier was applied to the training process to produce the best cell classification, which required multiple rounds of optimization. The trained classifier was then applied to all WSIs, and the number of cells and the area of each type were counted for quantification in the tumour parenchyma and stroma. The quality of cell segmentation and classification in the training course is of great importance for QuPath analysis, which was quality controlled by experienced pathologists.

### Multiplexed immunofluorescence

Six-color multiplex immunohistochemistry was performed using an OPAL Polaris system (Akoya Biosciences). Four-micrometer sections of FFPE tumours were routinely deparaffinized and hydrated. Heat-induced epitope retrieval in citrate buffer was performed before non-specific binding was blocked. Sections were sequentially stained with each primary antibody (anti-CK [1:1,500, Abcam, ab215838], anti-IDO1 [1:1,000, CST, #86630], anti-CD68 [1:600, Gen Tech, GM081429], anti-CD163 [1:800, Gen Tech, GT207729], and anti-PD-1 [1:200, CST, #43248]), corresponding horseradish peroxidase-conjugated secondary antibody, tyramide signal amplification, and OPAL fluorophore. OPAL 520 (CK), 570 (IDO1), 620 (PD-1), 690 (CD163), and 780 (CD68) dyes were used. The sections were then counterstained with spectral DAPI (Akoya Biosciences). A Vectra Polaris multispectral imaging system (Akoya Biosciences) was used to scan and image the fluorescence signals. The scanned images were annotated and visualized using PhenoChart (version 1.1.0, Akoya Biosciences), and analyzed using inForm software (version 2.5.0, Akoya Biosciences). The tumour parenchyma areas were identified using CK as a marker. CD68 and CD163 were used as pan-macrophage and M2 macrophage markers, respectively.

### Statistical analysis

The Mann-Whitney test and Wilcoxon matched-pairs signed rank test were used to compare differences between unpaired and paired two groups, respectively. The correlation between IDO1 expression and other relevant genes or the abundance of putative infiltrating immune cells was evaluated using Spearman correlation analysis. Statistical analysis was performed using R software (version 3.6.3) or GraphPad Prism (version 9.0.0). A *P*-value <0.05 was considered statistically significant. All statistical tests were two-sided.

## Results

### Survival analysis of IDO1 in patients with ESCC

**FIGURE 1:**
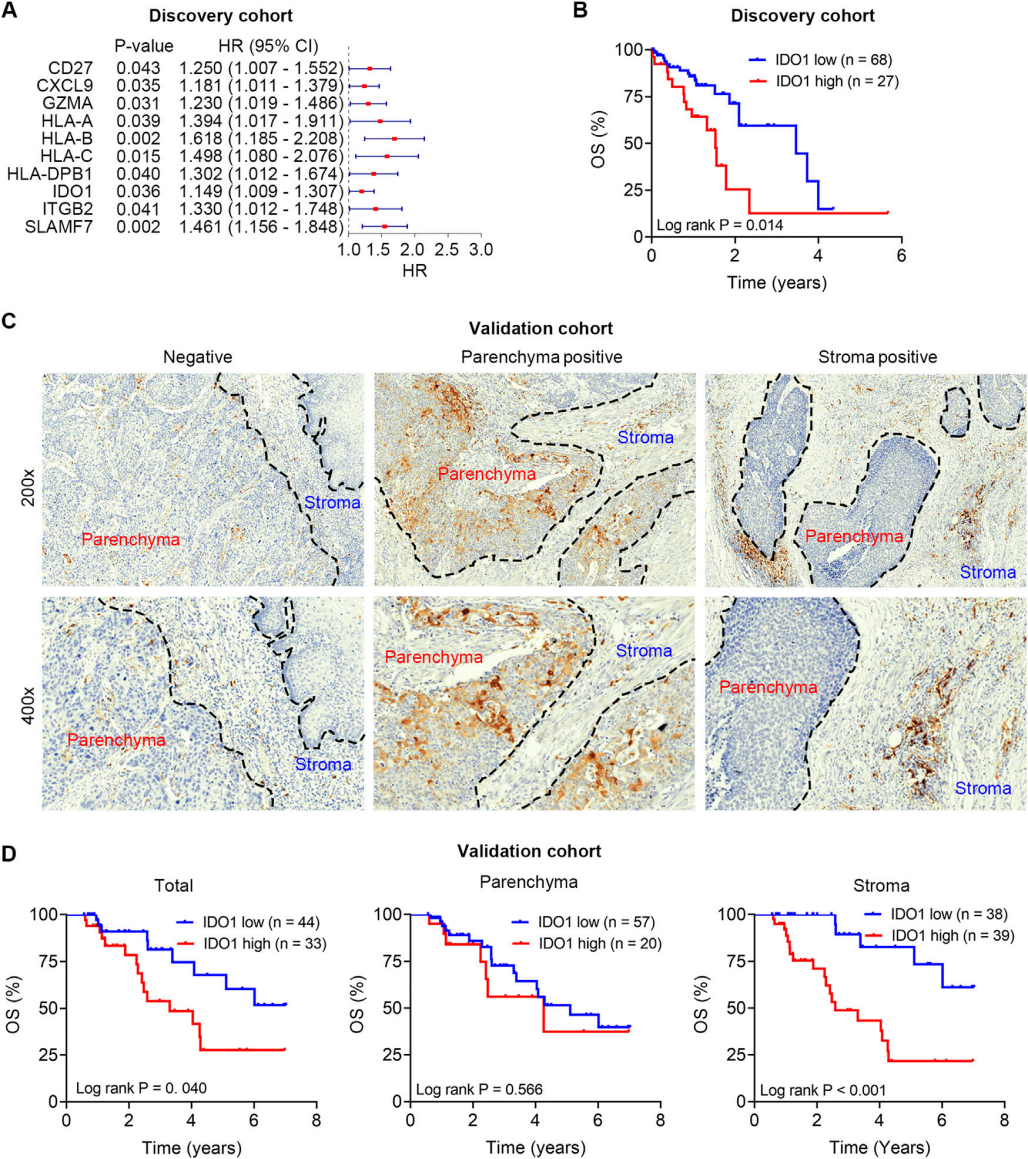
Survival analysis of IDO1 in the discovery and validation cohorts. **(A)** Univariate cox regression analysis to screen prognostic immunomodulators in the discovery cohort. **(B)** Kaplan-Meier analysis of ESCC patients’ OS in the discovery cohort (n = 95) stratified by IDO1 mRNA expression. **(C)** Representative immunohistochemistry micrographs of IDO1 protein expression in tumour parenchyma and stroma. **(D)** Kaplan-Meier analyses of ESCC patients’ OS in the validation cohort (n = 77) stratified by IDO1 protein expression level in total tumour (left panel), tumour parenchyma (middle panel), and tumour stroma (right panel). HR, hazard ratio; CI, confidential interval.

We first analyzed the prognostic importance of a panel of immunomodulators in TCGA ESCC dataset (n = 95), which was designated as the discovery cohort. Univariate Cox regression analysis revealed that the mRNA expression levels of CD27, CXCL9, GZMA, HLA-A, HLA-B, HLA-C, HLA-DPB1, IDO1, ITGB2, and SLAMF7 were significantly correlated with OS (all *P* < 0.05; Figure 1A).

Ample evidence suggests that IDO1 is an immunosuppressive molecule in TME, and therapeutic strategies targeting the Trp-IDO1-Kyn signaling using IDO1 inhibitors are currently being assessed in clinical trials; therefore, we focused on IDO1 in subsequent analyses. To further evaluate the prognostic value of IDO1, Kaplan-Meier survival analysis was used to examine the survival differences in ESCC patients with varying IDO1 expression. ESCC patients were divided into low- (n = 68) and high-IDO1 (n = 27) subgroups according to the optimal cut-off value of IDO1 expression levels in the Kaplan-Meier survival analysis. ESCC patients with higher IDO1 expression had significantly shorter OS (*P* = 0.014; Figure 1B).

Notably, the mRNA expression level does not always match the protein level; therefore, we detected IDO1 protein in the validation cohort using immunohistochemistry staining, and quantified its expression level in a digitalized manner using QuPath software. Heterogeneous protein expression of IDO1 was observed in different ESCC samples, even in different parts (parenchyma and stroma) within a certain sample (Figure 1C). Poor OS was observed in patients with high total IDO1 protein expression (*P* = 0.040; Figure 1D, left panel) and high stroma IDO1 protein expression (*P* < 0.001; Figure 1D, middle panel). However, no significant difference in OS was observed in patients with different parenchymal IDO1 protein expression (*P* = 0.566; Figure 1D, right panel).

### IDO1 is an independent prognostic indicator of OS in patients with ESCC

To further explore the clinical significance of IDO1 in ESCC, univariate Cox regression analysis with IDO1 expression and multiple clinicopathologic characteristics included was performed in the discovery cohort. The results showed that IDO1 mRNA (hazard ratio [HR] = 2.356, 95% confidence interval [CI]: 1.164 to 4.768, *P* = 0.017) and TNM stage (HR = 2.390, 95% CI: 1.165 to 4.903, *P* = 0.017) were significantly associated with OS in the discovery cohort. Multivariate Cox regression analysis showed that IDO1 mRNA (HR = 2.462, 95% CI: 1.188 to 5.099, *P* = 0.015) and TNM stage (HR = 2.221, 95% CI: 1.087 to 4.575, *P* = 0.030) were independent prognostic factors (Table 1). Above findings were confirmed in the validation cohort. Univariate Cox regression analysis showed that total IDO1 protein (HR = 2.484, 95% CI: 1.069 to 5.770, *P* = 0.034), stroma IDO1 protein (HR = 4.881, 95% CI: 1.805 to 13.196, *P* = 0.034), and TNM stage (HR = 3.363, 95% CI: 1.408 to 8.030, *P* = 0.006) were significantly associated with OS in the validation cohort. Multivariate Cox regression analysis showed that stroma IDO1 protein (HR = 3.539, 95% CI: 1.208 to 10.365, *P* = 0.021) and TNM stage (HR = 4.554, 95% CI: 1.723 to 12.041, *P* = 0.002) were independent prognostic factors (Table 2). Overall, these data suggest that IDO1 is an independent prognostic indicator of OS in patients with ESCC.

**TABLE 1:** Univariate and multivariate regression analysis of IDO1 mRNA expression in the discovery cohort.

Variables	Univariate regression		Multivariate regression	
	HR (95% CI)	*P*-value	HR (95% CI)	*P*-value
Age (<60 vs. ≥60)	1.710 (0.806–3.629)	0.162		
Gender (Female vs. Male)	3.866 (0.911–10.713)	0.062		
Grade (G1 + G2 vs. G3)	0.718 (0.271–1.903)	0.505		
TNM stage (I + II vs. III + IV)	2.390 (1.165–4.903)	**0.017**	2.221 (1.078–4.575)	**0.030**
IDO1 mRNA (Low vs. High)	2.356 (1.164–4.768)	**0.017**	2.462 (1.188–5.099)	**0.015**

HR, hazard ratio; CI, confidence interval; *P*-value bold if <0.05.

**TABLE 2:** Univariate and multivariate regression analysis of IDO1 protein expression in the validation cohort.

Variables	Univariate regression		Multivariate regression	
	HR (95% CI)	*P*-value	HR (95% CI)	*P*-value
Age (<60 vs. ≥ 60)	1.630 (0.727–3.656)	0.236		
Gender (Female vs. Male)	1.572 (0.697–3.542)	0.276		
Grade (G1+G2 vs. G3)	1.557 (0.642–3.774)	0.327		
TNM stage (I + II vs. III + IV)	3.363 (1.408–8.030)	**0.006**	4.554 (1.723–12.041)	**0.002**
Total IDO1 protein (Low vs. High)	2.484 (1.069–5.770)	**0.034**	2.171 (0.789–5.975)	0.133
Parenchyma IDO1 protein (Low vs. High)	1.361 (0.559–3.314)	0.497		
Stroma IDO1 protein (Low vs. High)	4.881 (1.805–13.196)	**0.002**	3.539 (1.208–10.365)	**0.021**

HR, hazard ratio; CI, confidence interval; *P*-value bold if <0.05.

### Association between IDO1 expression and immunological characteristics

**FIGURE 2:**
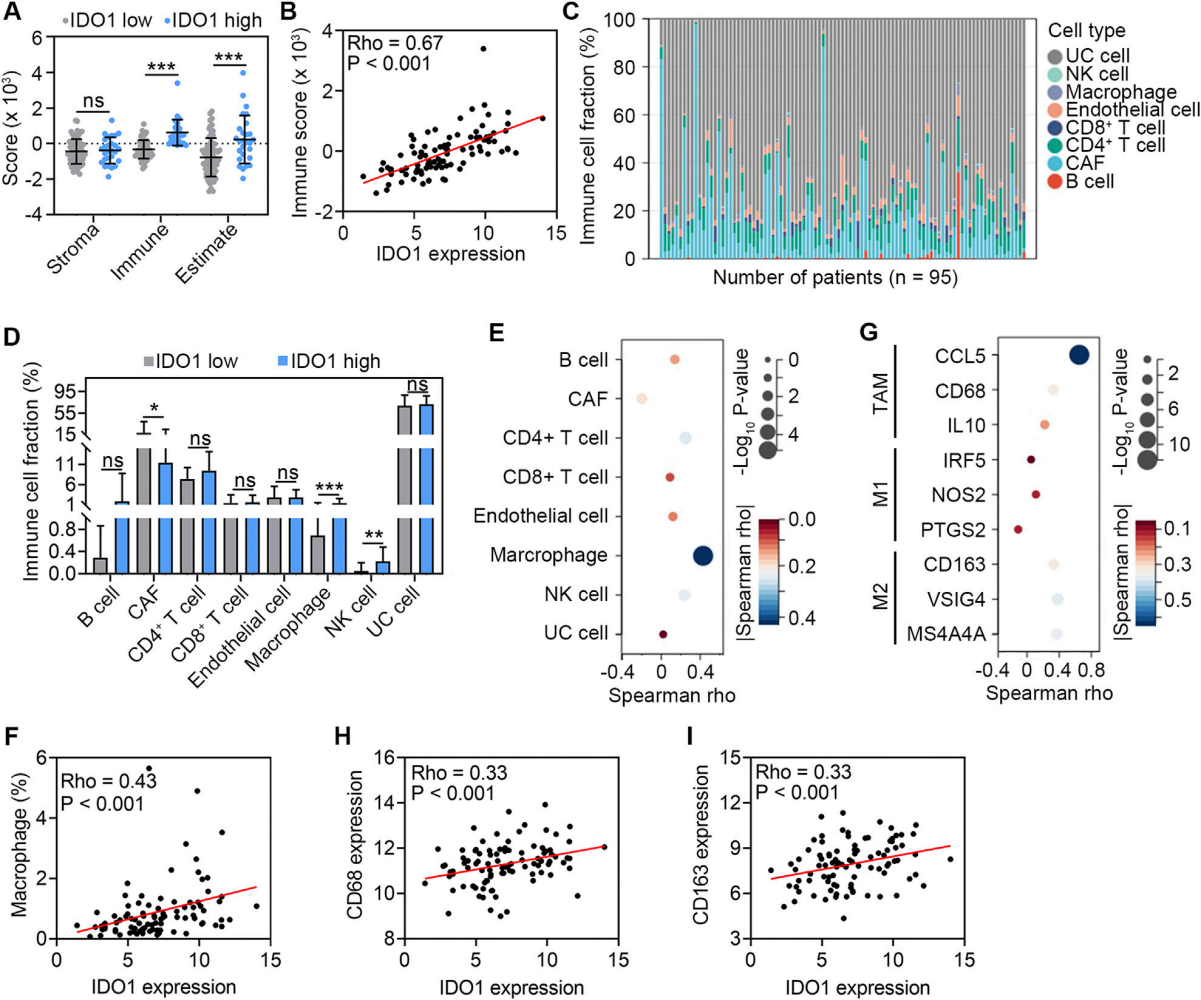
Association between IDO1 expression and immune cell abundance in TME. **(A)** Comparison of stroma score, immune score, and estimate score estimated by the ESTIMATE algorithm between ESCC patients with low (n = 68) and high (n = 27) IDO1 expression. **(B)** Scatter plot showing Spearman correlation between IDO1 mRNA expression and immune score. **(C)** Profile of eight types of cells in TME of ESCC samples (n = 95) estimated by the EPIC algorithm. **(D)** Comparison of the abundance of eight types of cells between ESCC patients with low (n = 68) and high (n = 27) IDO1 expression. **(E)** Bubble chart showing Spearman correlation between IDO1 mRNA expression and the abundance of eight types of cells. **(F)** Scatter plot showing Spearman correlation between IDO1 mRNA expression and the abundance of macrophages. **(G)** Bubble chart showing Spearman correlation between mRNA expression levels of IDO1 and marker genes of TAMs (CCL5, CD68, and IL10), M1 (IRF5, NOS2, and PTGS2), and M2 (CD163, VSIG4, and MS4A4A) macrophages. Scatter plot showing Spearman correlation between mRNA expression levels of IDO1 and CD68 **(H)**, IDO1 and CD163 **(I)** CAF, cancer-associated fibroblast; NK, nature killer; UC, uncharacterized cell. Values are means ± SD. Mann-Whitney test **(A, D)**. ns, no significance. *P < 0.05; **P < 0.01; ***P < 0.001.

Next, we explored the role of IDO1 in TME remodeling and immune cell regulation. According to the ESTIMATE algorithm, patients in the high-IDO1 subgroup had higher immune scores, indicating significantly higher infiltration of immune cells into TME (*P* < 0.001; Figure 2A). Correlation analysis also revealed a positive correlation between IDO1 expression and immune score (Rho = 0.67, *P* < 0.001; Figure 2B). Using the EPIC algorithm to calculate tumour-infiltrating immune cells (Figure 2C), we further investigated the difference in specific infiltrating immune cells between the two groups. The high-IDO1 subgroup had a significantly higher number of macrophages and NK cells and fewer CAFs (Figure 2D). Correlation analysis also revealed positive correlations between IDO1 expression and the number of macrophages and NK cells, and a negative correlation between IDO1 expression and the number of CAFs (Figure 2E). In particular, macrophages were most significantly correlated with IDO1 expression (Rho = 0.43, *P* < 0.001; Figure 2F). Therefore, we further explored the potential role of IDO1 in macrophage polarization. The relationship between IDO1 and marker genes of tumor-associated macrophages (TAMs; CCL5, CD68, and IL10), M1 (IRF5, NOS2, and PTGS2), and M2 (CD163, VSIG4, and MS4A4A) macrophages was analyzed. The Spearman correlation analysis showed that IDO1 was strongly correlated with marker genes of TAMs (CCL5 and CD68) and M2 macrophages (CD163, VSIG4, and MS4A4A) (all Rho > 0.3, all *P* < 0.001; Figures 2G–I). These results suggest that IDO1 may play a role in the regulation of macrophage polarization in ESSC.

**FIGURE 3:**
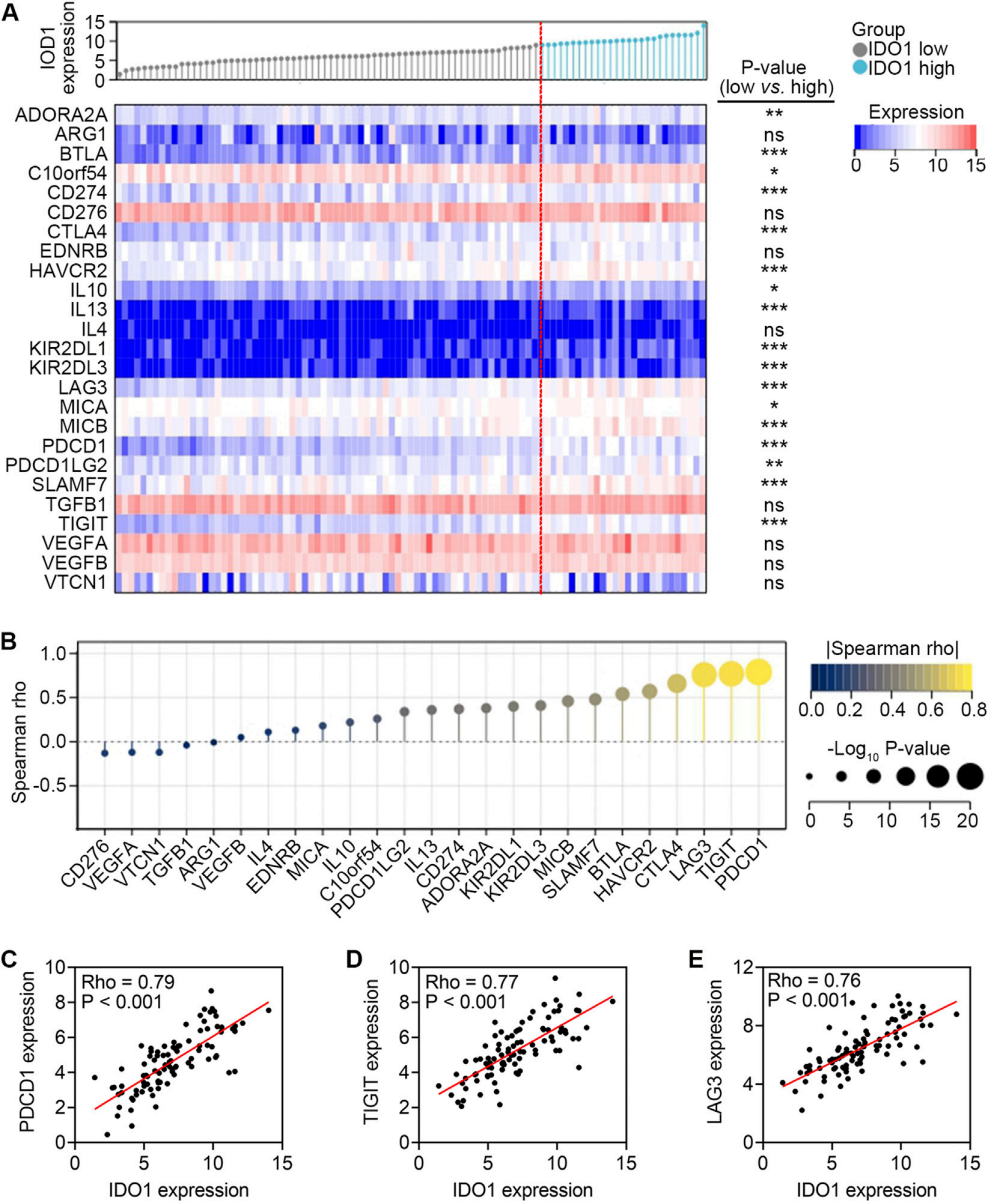
Association between mRNA expression levels of IDO1 and IC genes. **(A)** Comparison of mRNA expression levels of IC genes between ESCC patients with low (n = 68) and high (n = 27) IDO1 expression. **(B)** Lollipop chart showing Spearman correlation between mRNA expression levels of IDO1 and IC genes. Scatter plot showing Spearman correlation between mRNA expression levels of IDO1 and PDCD1 **(C)**, IDO1 and TIGIT **(D)**, IDO1 and LAG3 **(E)** Mann-Whitney test **(A)**. ns, no significance. *P < 0.05; **P < 0.01; ***P < 0.001.

In TME, ICs are co-inhibitors effectively engaged by tumour cells, immune cells, and stromal cells that bind to the ligands expressed on the cell surface of CD8^+^ T cells, triggering inhibitory signaling pathways and leading to the quiescence or exhaustion of CD8^+^ T cells (Palucka and Coussens, 2016). Patients in the high-IDO1 subgroup had higher expression of a range of ICs, including ADORA2A, BTLA, C10orf54, CD274, CTLA4, HAVCR2, IL10, IL13, KIR2DL1, KIR2DL3, LAG3, MICA, MICB, PDCD1, PDCD1LG2, SLAMF7, and TIGIT (all *P* < 0.05; Figure 3A). Additionally, the correlation analysis revealed that IDO1 had a strong correlation with ADORA2A, BTLA, CD274, CTLA4, HAVCR2, IL13, KIR2DL1, KIR2DL3, LAG3, MICB, PDCD1, PDCD1LG2, SLAMF7 and TIGIT (all Rho > 0.3, all *P* < 0.001; Figure 3B), among which PDCD1, TIGIT, and LAG3 were most positively correlated with IDO1 (Figures 3C–E). These results imply that ESCC with high IDO1 expression possesses a suppressive TME.

### Coexpression of IDO1 and PD-1 on macrophages

**FIGURE 4:**
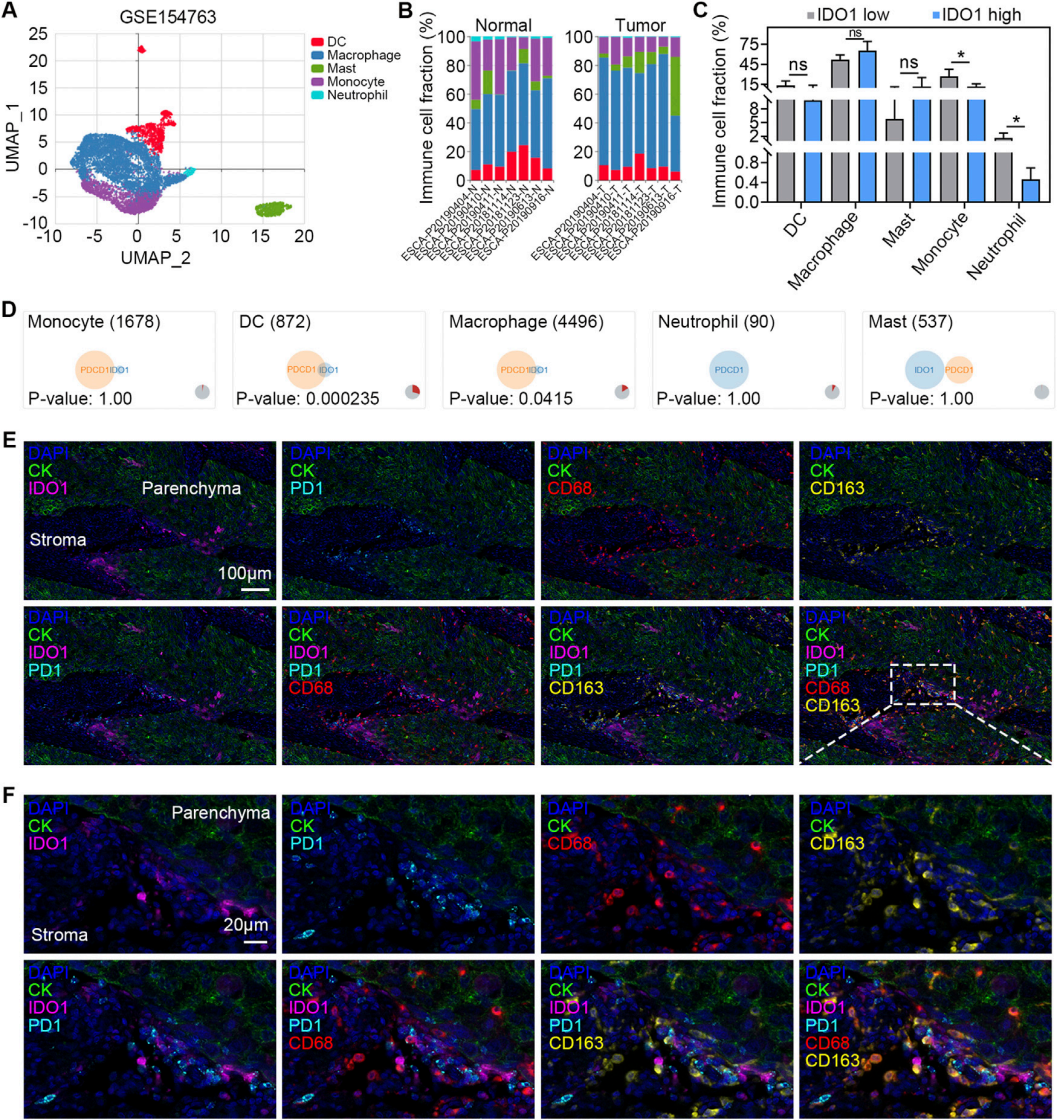
Coexpression of IDO1 and PD-1 on macrophages. **(A)** UMAP plot of GSE154763 scRNA-seq dataset. The cells are colored according to their immune annotation. **(B)** Bar plots of the percentage of cells per cell types in tumour (n = 6) and adjacent normal (n = 6) samples. **(C)** Paired comparison of the percentage of cells per cell type in tumour (n = 6) and adjacent normal (n = 6) samples. **(D)** Venn diagram showing the coexpression of IDO1 and PDCD1 in different types of immune cells. Representative multiplexed immunofluorescence micrographs **(E)** and the detail with enlarged scale **(F)** showing expression of IDO1 (pink), PD-1 (cyan), CD68 (red), and CD163 (yellow) in tumour parenchyma and stroma. DAPI (blue) and CK (green) were used for nuclear and tumour parenchyma staining, respectively. Scale bar = 100 μm in **(E)**, and 20 μm in **(F)**. Values are means ± SD. Wilcoxon matched-pairs signed rank test **(C)**. ns, no significance. *P < 0.05.

In the above analyses, we found that IDO1 expression was positively correlated with the number of macrophages in TME (Figure 2F), as well as the expression of PDCD1 within a range of ICs (Figure 3C), prompting us to further investigate the cellular distribution of IDO1 and PDCD1 in macrophages. We performed scRNA-seq data analysis using the IMMUcan scDB database. GSE154763 containing seven pairs of esophageal tumours and adjacent tissues was searched and selected. Five cell clusters (dendritic cells [DCs], macrophages, mast cells, monocytes, and neutrophils) within the GSE154763 were dissected by scRNA-seq analysis (Figure 4A). The cell compositions of ESCC samples and adjacent tissues are shown in Figure 4B. Monocytes and neutrophils were significantly downregulated in ESCC samples compared with those in adjacent tissues (both *P* < 0.05; Figure 4C), whereas no difference was found in cell fractions of DCs, macrophages, and mast cells (all *P* > 0.05; Figure 4C). Coexpression analysis revealed a highly significant overlap of IDO1 and PDCD1 in DCs (*P* < 0.001; Figure 4D) and macrophages (*P* < 0.05; Figure 4D), but not in monocytes, mast cells, and neutrophils (all *P* > 0.05; Figure 4D). To further validate the results of scRNA-seq analyses, we conducted multiplexed immunofluorescence to analyze IDO1 and PD-1 expression in TME. We observed colocalization of IDO1, PD-1, CD68 (marker of TAMs), and CD163 (marker of M2 macrophages) in the tumour stroma (Figures 4E, F). In aggregate, these data suggest that IDO1 and PD-1 are coexpressed on macrophages in TME of ESSC.

### Association between IDO1 expression and therapeutic response

**FIGURE 5:**
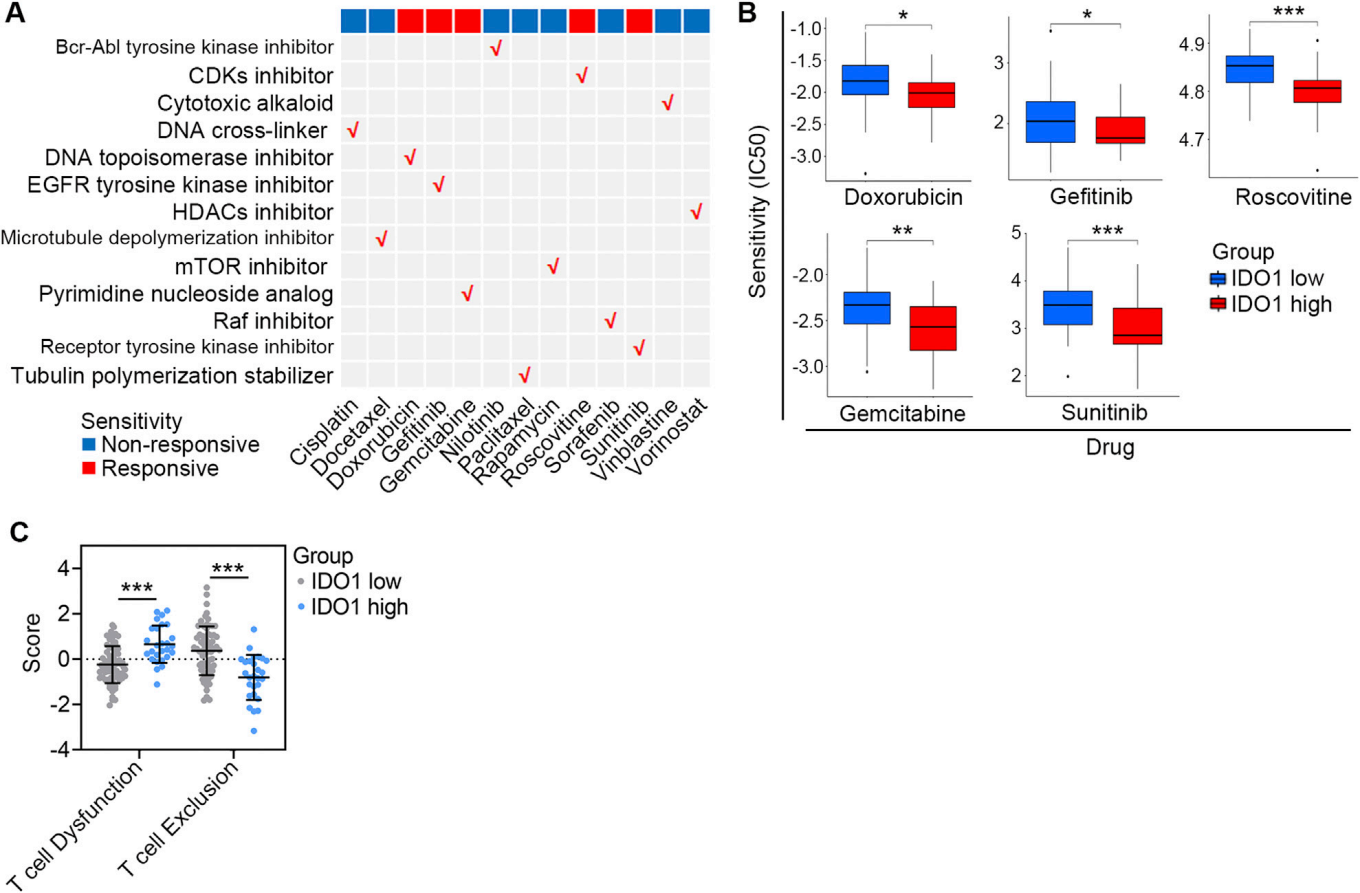
Association between IDO1 expression and therapeutic response. **(A)** Summary of the sensitivity of chemotherapeutic drugs with different mechanisms in ESCC patients with disparate IDO1 expression. **(B)** Bar plots of the IC50 in IDO1 low (n = 68) and high (n = 27) ESCC patients. **(C)** Comparison of T cell dysfunction and T cell exclusion scores estimated by the TIDE algorithm between ESCC patients with low (n = 68) and high (n = 27) IDO1 expression. IC50, half maximal inhibitory concentration. Values are means ± SD. Mann-Whitney test **(B, C)**. *P < 0.05; **P < 0.01; ***P < 0.001.

The differences of drug sensitivity between the low- and high-IDO1 subgroups in 13 commonly used chemotherapeutic medications (Figure 5A) were analyzed using the “pRRophetic” R package. The high-IDO1 subgroup showed significantly increased sensitivity to doxorubicin (*P* < 0.05), gefitinib (*P* < 0.05), gemcitabine (*P* < 0.01), roscovitine (*P* < 0.001), and sunitinib (*P* < 0.001) (Figure 5B).

Two stages of T cell dysfunction have been found, and anti-PD-1 treatment can deteriorate early stage dysfunctional T cells; however, late-stage dysfunctional T cells are resistant to IC blockage reprogramming (Philip et al., 2017). TIDE dysfunction signatures were able to infer dysfunctional T cells in the late stages based on gene expression profiles (Jiang et al., 2018); therefore, we used the TIDE algorithm to estimate T cell dysfunction in patients. Compared with those in the low-IDO1 subgroup, patients in the high-IDO1 subgroup had higher T-cell dysfunction but lower T-cell exclusion scores (both *P* < 0.001; Figure 5C), reflecting the profiles of dysfunctional T cells in the late stages. Collectively, these data indicate that ESSC patients with higher IDO1 expression show increased sensitivity to a range of chemotherapeutic drugs, while are more likely to resist IC inhibitor administration.

### Exploring the signaling pathways associated with IDO1

**FIGURE 6:**
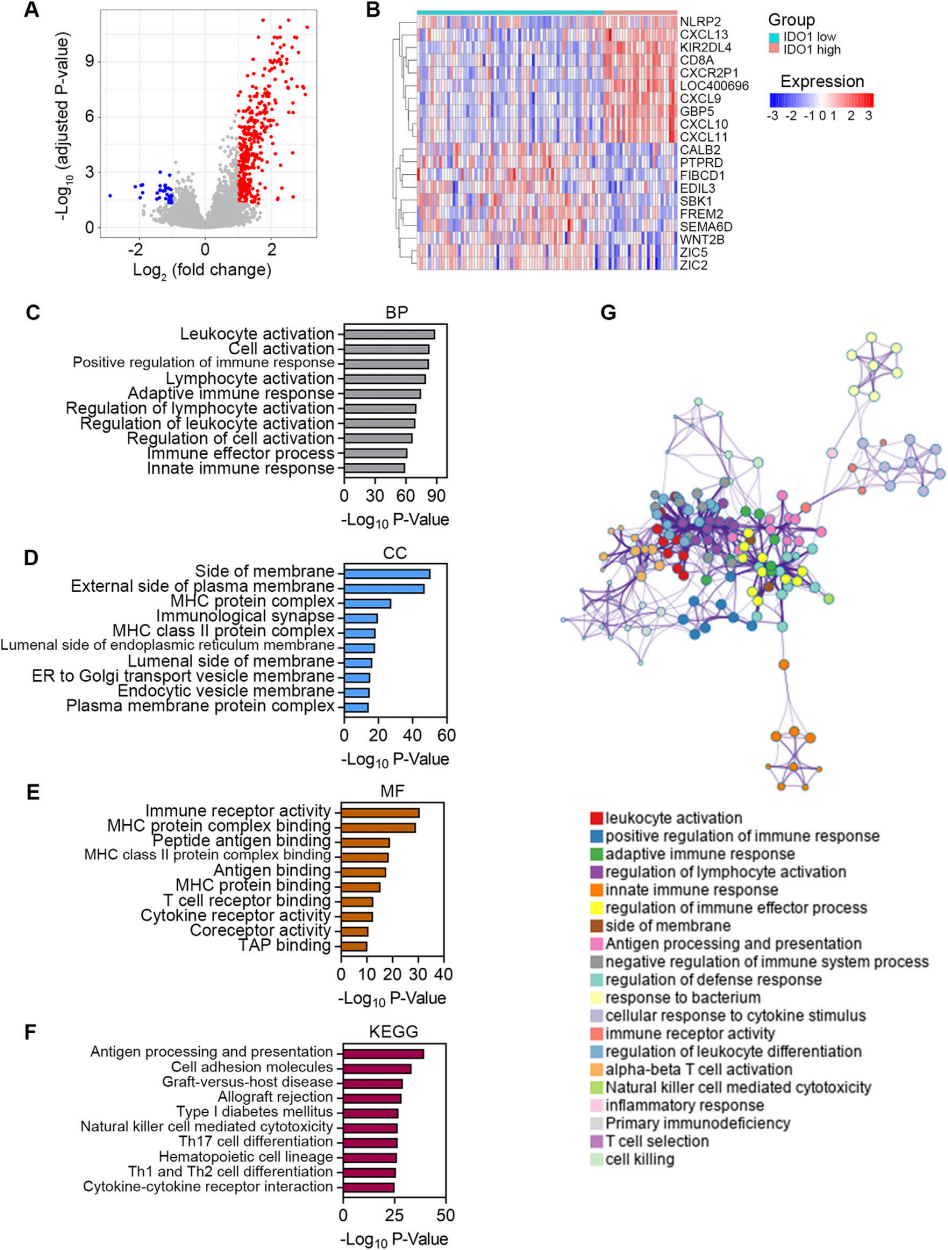
Exploring the signaling pathways associated with IDO1. **(A)** Volcano plot showing DEGs between ESCC patients with low (n = 68) and high (n = 27) IDO1 expression. The screening criteria were set as adjusted *P* < 0.05 and | log_2_ (fold change) | ≥ 1. **(B)** Expression profile of top 10 DEGs. Bar plots showing top 10 significantly enriched terms related to BP **(C)**, CC **(D)**, and MF **(E)** based on GO analysis. **(F)** Bar plot showing top 10 significantly enriched terms based on KEGG analysis. **(G)** Network plot showing relationships between the enriched terms. Nodes represent enriched gene sets that are grouped and annotated by their similarity according to related gene sets. Node size is proportional to the total number of genes within each gene set. Proportion of shared genes between gene sets is represented as the thickness of the connecting line between nodes. BP, Biological Process; CC, Cellular Component; MF, Molecular Function; KEGG, Kyoto Encyclopedia of Genes and Genomes.

Finally, we explored the signaling pathways associated with IDO1 to elucidate the possible underlying molecular mechanisms of IDO1 in ESCC. Differential analysis using the “limma” R package revealed a total of 440 DEGs between low- and high-IDO1 subgroups, with 403 genes upregulated and 37 genes downregulated (Figure 6A). The expression profiles of the top 10 dysregulated DEGs are shown in Figure 6B. These DEGs were further subjected to Metascape for signaling pathway exploration. Genes enriched for biological processes in GO analysis were mainly associated with leukocyte activation and innate and adaptive immune responses (top 10 terms shown in Figure 6C). Genes enriched for cellular components in the GO analysis were principally associated with side of membrane, vesicle membrane, and MHC protein complex (top 10 terms shown in Figure 6D). Genes enriched for molecular functions in GO analysis were predominantly associated with immune receptor activity, MHC protein binding, and cytokine receptor activity (top 10 terms shown in Figure 6E). Genes enriched in KEGG analysis were generally associated with antigen processing and presentation, cell adhesion molecules, and cytokine-cytokine receptor interactions (top 10 terms shown in Figure 6F). These enriched terms were further classified into 20 clusters based on their membership similarities (Figure 6G).

## Discussion

In recent years, cancer immunotherapy has emerged as a groundbreaking approach, and the inhibition of IDO1 has been explored as a potential strategy (Charehjoo et al., 2023). Despite initial promise, clinical trials in this area have faced challenges (Peyraud et al., 2022). Our research addresses this issue by shifting the focus toward the role of macrophages in TME as potential therapeutic targets. Based on the analysis of RNA sequencing data from 95 patients with ESCC from TCGA database, our study delved into the prognostic significance of IDO1. Utilizing a range of bioinformatics tools, we not only established the association of IDO1 with poor patient survival but also highlighted its independent prognostic value. We validated the expression of IDO1 in the stroma and parenchyma of tumour samples from our clinical center in a computer-guided digital manner with QuPath software. Moreover, we comprehensively explored TME of ESSC through estimating scores for stromal and immune cells, and assessing the abundance of various immune cell types within TME. Our findings demonstrated a positive correlation between IDO1 expression and macrophages in TME. Furthermore, our scRNA-seq data analysis and multiplexed immunofluorescence revealed a compelling connection between IDO1 and PD-1, particularly coexpressed on TAMs. This observation opens new avenues for potential targets in ESCC immunotherapy. We also explored the sensitivity of patients with high IDO1 expression to chemotherapeutic drugs and their resistance to immune checkpoint blockade, providing valuable insights for future clinical strategies.

TME is a complex and dynamic entity that has been extensively implicated in tumourignesis. It harbors tumour cells that interact with surrounding cells, especially immune cells, to influence tumour growth, metastasis, and response to therapy. Previous studies have demonstrated the immunomodulatory effects of IDO1 on multiple types of immune cells, including tumor-associated DCs, regulatory T cells (Tregs), myeloid-derived suppressor cells (MDSCs), NK cells, and TAMs. In particular, TAMs are the most abundant cell types in solid tumours and usually exhibit an M2-like phenotype that participates in tumour immunosuppression and leads to the immune escape of cancer cells. IDO1 expressing immune cells, especially macrophages, were found to be more abundant in malignant tissues and associated with worse prognosis of many cancer types, such as penile squamous cell carcinoma (Zhou et al., 2020), oral squamous cell carcinoma of advanced stages (Struckmeier et al., 2023), and classical Hodgkin lymphoma (Karihtala et al., 2020). Moreover, increased numbers of IDO1^+^ TAMs in breast cancer patients upregulated pro-tumourigenic factors associated with resistance to immunosuppressive therapy after anti-PD-1 treatment (Chang et al., 2024). The results in our study were complied with above findings, adding fundamental evidences to the combination of IDO1 inhibition and IC blockage for cancer treatment, though the much-anticipated phase III clinical trial (ECHO-301/KEYNOTE-252) of IDO1 inhibitor compound **29** combined with PD-1 inhibitor failed (Long et al., 2019). Future research should focus on comprehensive understanding of the role of IDO1 in TME, especially the immunosuppression caused by IDO1^+^ TAMs in immunotherapy.

## Limitations

This study has several limitations. With the convenience of large-scale datasets, bioinformatics offers valuable insights into cancer research; however, the introduction of big data analytics inevitably produces unique biases and sources of variation, which need to be carefully considered and addressed. Consequently, bioinformatics results may not precisely mirror the effect observed in practical clinical settings (Baykal et al., 2024).

Another limitation arises from the diversity and the relatively small sample size of the study cohorts. The discovery cohort (n = 95) is collected from TCGA database that contains individuals of differing races, while the validation cohort (n = 77) population is recruited from our clinical center consisting solely of East Asians. The discrepancy in population backgrounds and the relatively insufficient participants could result in potential bias.

It is noteworthy that prognosis is influenced by multiple factors, including specific genetic profiles, tumour stages, and treatment modalities. Consequently, survival outcomes may be affected by other potential confounding variables that were not incorporated into the prognostic analysis. In the context of the study’s focus on chemotherapy and immunotherapy, the therapeutic response in both the discovery and validation datasets may serve as a more pertinent phenotype than survival. However, it is a fact that the follow-up information of treatment response in the discovery and validation cohorts is incomplete.

Moreover, treatment response was initially assessed using computational approaches. While this approach identifies potential drug targets, it does not guarantee their effectiveness in clinical settings. Experimental validation and clinical trials are necessary to confirm the findings.

Finally, there is few experimental data regarding the role of IDO1 in macrophages. To this end, subsequent experimental exploration is necessary to unveil the function and molecular mechanism of IDO1 in macrophages.

## Suggestions for future research directions

In light of the study’s limitations, several suggestions can guide future research directions in this critical area. Firstly, future investigations should aim to compose a set of recommended standards and guidelines aimed at promoting reproducibility in bioinformatics, and enhancing translation of bioinformatics findings into medical practice.

Secondly, to improve the generalizability of our findings, future studies should include a larger patient cohorts consisting of individuals with different genetic backgrounds. In addition, more detailed clinicopathological characteristics should be recoded on file.

Lastly, it is important to note that therapeutic response achieved through *in silico* prediction does not guarantee their effectiveness in clinical settings. Furthermore, the role of IDO1 in macrophages remained unveiled. Therefore, experimental explorations and clinical trials should be designed to address above issues.

## Conclusion

In conclusion, this research integrated bioinformatics analyses and digital pathology assessment to identify IDO1 as an independent prognostic indicator of OS in patients with ESCC. Moreover, our research revealed a compelling connection between IDO1 and PD-1, particularly coexpressed on TAMs through comprehensive exploration of TME and multiplexed immunofluorescence validation in tumour samples. We also explored the sensitivity of patients with high IDO1 expression to chemotherapeutic drugs and their resistance to IC blockade. Our study contributes to the understanding of the complex interplay of IDO1, PD-1, and macrophages in TME of ESCC. These observations open new avenues for potential targets in ESCC immunotherapy. Further research and clinical trials are needed to explore the role IDO1 in macrophages, and to elaborate the clinical findings of the present study.

## Data Availability

The original contributions presented in the study are included in the article/Supplementary Material, further inquiries can be directed to the corresponding authors.

## Abbreviations

IDO1, Indolamine-2,3-dioxygenase 1; TME, tumor microenvironment; ESCC, esophageal squamous cell carcinoma; TCGA, The Cancer Genome Atlas; IC, immune checkpoint; PD-1, programmed cell death 1; PD-L1, programmed cell death 1 ligand 1; OS, overall survival; FFPE, formalin-fixed paraffin-embedded; CAF, cancer associated fibroblast; scRNA-seq, single-cell RNA sequencing; TIDE, Tumor Immune Dysfunction and Exclusion; DEGs, differentially expressed genes; GO, Gene Ontology; KEGG, Kyoto Encyclopedia of Genes and Genomes; WSI, whole-slide image; HR, hazard ratio; CI, confidence interval; TAMs, tumor-associated macrophages; DCs, dendritic cells; Tregs, regulatory T cells; MDSCs, myeloid-derived suppressor cells; IC50, half-maximal inhibitory concentration.
