# Supplementary material for: Integrated analyses reveal IDO1 as a prognostic biomarker coexpressed with PD-1 on tumor-associated macrophages in esophageal squamous cell carcinoma
Source: Front Pharmacol. 2024 Sep 16;15:1466779. doi: 10.3389/fphar.2024.1466779 (PMC11439782; doi:10.3389/fphar.2024.1466779)
Supplement: Supplementary file 1 [file Table1.docx]

**Table S1.** Baseline clinical characteristics of ESSC patients included in the discovery and validation cohorts

| Variables | Discovery cohort (total n = 95) | validation cohort (total n = 77) |
| --- | --- | --- |
| **Age** |  |  |
| < 60 | 57 (60%) | 49 (63.64%) |
| ≥ 60 | 38 (40%) | 28 (36.36%) |
| **Gender** |  |  |
| Male | 81 (85.26%) | 53 (68.83%) |
| Female | 14 (14.74%) | 24 (31.17%) |
| **Tumor grade** |  |  |
| G1 | 16 (16.84%) | 8 (10.39%) |
| G2 | 48 (50.53%) | 55 (71.43%) |
| G3 | 21 (22.11%) | 14 (18.18%) |
| NA | 10 (10.52%) | 0 (0%) |
| **TNM stage** |  |  |
| Ⅰ | 7 (7.37%) | 4 (5.19%) |
| Ⅱ | 55 (57.89%) | 33 (42.86%) |
| Ⅲ | 27 (28.42%) | 33 (42.86%) |
| Ⅳ | 4 (4.21%) | 7 (9.09%) |
| NA | 2 (2.11%) | 0 (0%) |
| **Survival status** |  |  |
| Alive | 63 (66.32%) | 53 (38.83%) |
| Dead | 32 (33.68%) | 24 (31.17%) |

Data are n (%). NA, not available
