# Supplementary material for: Integrated analyses reveal IDO1 as a prognostic biomarker coexpressed with PD-1 on tumor-associated macrophages in esophageal squamous cell carcinoma
Source: Front Pharmacol. 2024 Sep 16;15:1466779. doi: 10.3389/fphar.2024.1466779 (PMC11439782; doi:10.3389/fphar.2024.1466779)
Supplement: Supplementary file 2 [file Table2.docx]

**Table S2.** The list of 78 immunomodulators analyzed in this study

| **HGNC symbol** | **Friendly name** | **Gene family** | **Immune checkpoint** | **Super category** |
| --- | --- | --- | --- | --- |
| ADORA2A | ADORA2A | Receptor | Inhibitory | Receptor |
| ARG1 | ARG1 | Enzyme | Inhibitory | Other |
| BTLA | BTLA | Immunoglobulin | Inhibitory | Receptor |
| BTN3A1 | BTN3A1 | Butyrophilins | Stimulatory | Co-inhibitor |
| BTN3A2 | BTN3A2 | Butyrophilins | Stimulatory | Co-inhibitor |
| CCL5 | RANTES | Chemokine | Stimulatory | Ligand |
| CD27 | CD27 | TNFR | Stimulatory | Receptor |
| CD274 | PD-L1 | B7/CD28 | Inhibitory | Co-inhibitor |
| CD276 | CD276 | B7/CD28 | Inhibitory | Co-inhibitor |
| CD28 | CD28 | B7/CD28 | Stimulatory | Co-stimulator |
| CD40 | CD40 | TNFR | Stimulatory | Receptor |
| CD40LG | CD40LG | TNF | Stimulatory | Ligand |
| CD70 | CD70 | TNF | Stimulatory | Ligand |
| CD80 | CD80 | B7/CD28 | Stimulatory | Co-stimulator |
| CTLA4 | CTLA4 | Receptor | Inhibitory | Receptor |
| CX3CL1 | CX3CL1 | CXC chemokine | Stimulatory | Ligand |
| CXCL10 | IP-10 | CXC chemokine | Stimulatory | Ligand |
| CXCL9 | CXCL9 | CXC chemokine | Stimulatory | Ligand |
| EDNRB | EDNRB | Receptor | Inhibitory | Receptor |
| ENTPD1 | ENTPD1 | Ectonucleotidase | Stimulatory | Other |
| GZMA | GZMA | Granzyme | Stimulaotry | Other |
| HAVCR2 | TIM-3 | Immunoglobulin | Inhibitory | Receptor |
| HLA-A | HLA-A | MHC Class I |  | Antigen presentation |
| HLA-B | HLA-B | MHC Class I |  | Antigen presentation |
| HLA-C | HLA-C | MHC Class I |  | Antigen presentation |
| HLA-DPA1 | HLA-DPA1 | MHC Class II |  | Antigen presentation |
| HLA-DPB1 | HLA-DPB1 | MHC Class II |  | Antigen presentation |
| HLA-DQA1 | HLA-DQA1 | MHC Class II |  | Antigen presentation |
| HLA-DQA2 | HLA-DQA2 | MHC Class II |  | Antigen presentation |
| HLA-DQB1 | HLA-DQB1 | MHC Class II |  | Antigen presentation |
| HLA-DQB2 | HLA-DQB2 | MHC Class II |  | Antigen presentation |
| HLA-DRA | HLA-DRA | MHC Class II |  | Antigen presentation |
| HLA-DRB1 | HLA-DRB1 | MHC Class II |  | Antigen presentation |
| HLA-DRB3 | HLA-DRB3 | MHC Class II |  | Antigen presentation |
| HLA-DRB4 | HLA-DRB4 | MHC Class II |  | Antigen presentation |
| HLA-DRB5 | HLA-DRB5 | MHC Class II |  | Antigen presentation |
| HMGB1 | HMGB1 | HMG-box | Stimulatory | Other |
| ICAM1 | ICAM1 |  | Stimulatory | Cell adhesion |
| ICOS | ICOS | B7/CD28 | Stimulatory | Receptor |
| ICOSLG | ICOSLG | Ligand | Stimulatory | Co-stimulator |
| IDO1 | IDO1 | Enzyme | Inhibitory | Other |
| IFNA1 | IFNA1 | Cytokine | Stimulatory | Ligand |
| IFNA2 | IFNA2 | Cytokine | Stimulatory | Ligand |
| IFNG | IFNG | Cytokine | Stimulatory | Ligand |
| IL10 | IL10 | Cytokine | Inhibitory | Ligand |
| IL12A | IL12 | Cytokine | Stumulatory | Ligand |
| IL13 | IL13 | Cytokine | Inhibitory | Ligand |
| IL1A | IL1A | Cytokine | Stimulatory | Ligand |
| IL1B | IL1B | Cytokine | Stimulatory | Ligand |
| IL2 | IL2 | Cytokine | Stimulatory | Ligand |
| IL2RA | IL2RA | Type I cytokine receptor | Stimulatory | Receptor |
| IL4 | IL4 | Cytokine | Inhibitory | Ligand |
| ITGB2 | ITGB2 | Integrin | Stimulatory | Cell adhesion |
| KIR2DL1 | KIR2DL1 | KIR | Inhibitory | Receptor |
| KIR2DL2 | KIR2DL2 | KIR | Inhibitory | Receptor |
| KIR2DL3 | KIR2DL3 | KIR | Inhibitory | Receptor |
| LAG3 | LAG3 | Immunoglobulin | Inhibitory | Receptor |
| MICA | MICA | MHC Class I |  | Antigen presentation |
| MICB | MICB | MHC Class I |  | Antigen presentation |
| PDCD1 | PD-1 | B7/CD28 | Inhibitory | Receptor |
| PDCD1LG2 | PD-L2 | B7/CD28 |  | Co-inhibitor |
| PRF1 | PRF1 | Pore | Stimulatory | Other |
| SELP | SELP | Selectin | Stimulatory | Cell adhesion |
| SLAMF7 | SLAMF7 | SLAM | Inhibitory | Co-inhibitor |
| TGFB1 | TGFB1 | Cytokine | Inhibitory | Ligand |
| TIGIT | TIGIT | PVR | Inhibitory | Receptor |
| TLR4 | TLR4 | Receptor | Stimulatory | Receptor |
| TNF | TNF | Cytokine | Stimulatory | Ligand |
| TNFRSF14 | HVEM | TNFR | Stimulatory | Receptor |
| TNFRSF18 | TNFRSF18 | TNFR | Stimulatory | Receptor |
| TNFRSF4 | OX40 | TNFR | Stimulatory | Receptor |
| TNFRSF9 | 4-1BB | TNFR | Stimulatory | Receptor |
| TNFSF4 | OX40L | TNF | Stimulatory | Ligand |
| TNFSF9 | 4-1BB-L | TNF | Stimulatory | Ligand |
| VEGFA | VEGFA | Growth factor | Inhibitory | Ligand |
| VEGFB | VEGFB | Growth factor | Inhibitory | Ligand |
| C10orf54 | VISTA | Immunoglobulin | Inhibitory | Co-inhibitor |
| VTCN1 | VTCN1 | B7/CD28 | Inhibitory | Co-inhibitor |
